# Supplementary material for: Construction of Tongue Image-Based Machine Learning Model for Screening Patients with Gastric Precancerous Lesions
Source: J Pers Med. 2023 Jan 31;13(2):271. doi: 10.3390/jpm13020271 (PMC9968136; doi:10.3390/jpm13020271)

# Supplementary

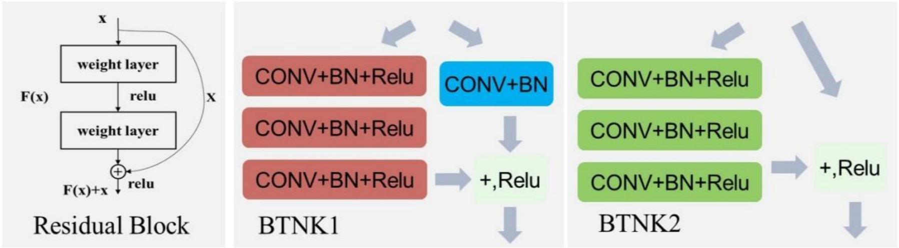


**Figure S1.** The residual module of the ResNet50 model.

**Table S1**. Univariate and multivariate analysis of symptoms factors in PLGC screening.

| **Variable** | **Total (N = 1995) No. (%)** | **Non-PLGC (N = 1824) No. (%)** | **PLGC (N = 171) No. (%)** | **P value*** | **Adjusted OR (95% Ci) †** | **P value#** |
|---|---|---|---|---|---|---|
| Symptoms Factors | | | | | | |
| Xerostomia | | | | $5.9 \times 10^{-6}$ | | |
| Yes | 642(0.32) | 560(0.31) | 82(0.48) | | 2.07([1.41,3.04]) | $1.9 \times 10^{-4}$ |
| No | 1353(0.68) | 1264(0.69) | 89(0.52) | | | |
| Bitter Taste | | | | $2.7 \times 10^{-2}$ | | |
| Yes | 412(0.21) | 365(0.20) | 47(0.27) | | 1.54([1.01,2.36]) | $4.7 \times 10^{-2}$ |
| No | 1583(0.79) | 1459(0.80) | 124(0.73) | | | |
| Belching | | | | $2.7 \times 10^{-2}$ | | |
| Yes | 518(0.26) | 461(0.25) | 57(0.33) | | 1.72([1.15,2.56]) | $7.9 \times 10^{-3}$ |
| No | 1477(0.74) | 1363(0.75) | 114(0.67) | | | |

P value* refer to the univariate analysis. P value# refer to the multivariate analysis (with adjustment for gender and age).

†Variables without significance (p>0.05) are not shown.

Table S2. Univariate analysis of tongue diagnostic and symptoms factors in PLGC screening.

| **Variable** | **Total (N = 1995) No. (%)** | **Non-PLGC (N = 1824) No. (%)** | **PLGC (N = 171) No. (%)** | **P value** | **OR (95% Ci)** |
|---|---|---|---|---|---|
| Tongue Diagnostic Labels | | | | | |
| Teeth Marks | | | | 0.49 | |
| Yes | 347(0.17) | 321(0.18) | 26(0.15) | | 0.84([0.54,1.30]) |
| No | 1648(0.83) | 1503(0.82) | 145(0.85) | | |
| Spotted | | | | 0.51 | |
| Yes | 29(0.01)) | 28(0.02) | 1(0.01) | | 0.38([0.06,2.58]) |
| No | 1966(0.99) | 1796(0.98) | 170(0.99) | | |
| Symptoms Factors | | | | | |
| Stomach Dull Pain | | | | 0.17 | |
| Yes | 273(0.14) | 256(0.14) | 17(0.10) | | 0.68([0.40,1.13]) |
| No | 1722(0.86) | 1568(0.86) | 154(0.90) | | |
| Constipated | | | | 0.20 | |

| | | | | | |
|---|---|---|---|---|---|
| Yes | 104(0.05) | 91(0.05) | 13(0.08) | | 1.57([0.86,2.85]) |
| No | 1891(0.95) | 1733(0.95) | 158(0.92) | | |
| Stomach Bloating | | | | 0.33 | |
| Yes | 1491(0.75) | 1369(0.75) | 122(0.71) | | 0.83([0.58,1.17]) |
| No | 504(0.25) | 455(0.25) | 49(0.29) | | |
| Belching | | | | 0.38 | |
| Yes | 1108(0.56) | 1019(0.56) | 89(0.52) | | 0.86([0.63,1.17]) |
| No | 887(0.44) | 805(0.44) | 82(0.48) | | |
| Loose Stools | | | | 0.60 | |
| Yes | 53(0.03) | 50(0.03) | 3(0.02) | | 0.63([0.20,2.03]) |
| No | 1942(0.97) | 1774(0.97) | 168(0.98) | | |
| Stomach Stinging | | | | 0.60 | |
| Yes | 66(0.03) | 62(0.03) | 4(0.02) | | 0.68([0.25,1.88]) |
| No | 1929(0.97) | 1762(0.97) | 167(0.98) | | |
| Gastric Cardialgia | | | | 1.0 | |
| Yes | 38(0.02) | 35(0.02) | 3(0.02) | | 0.91([0.28,3.00]) |
| No | 1957(0.98) | 1789(0.98) | 168(0.98) | | |

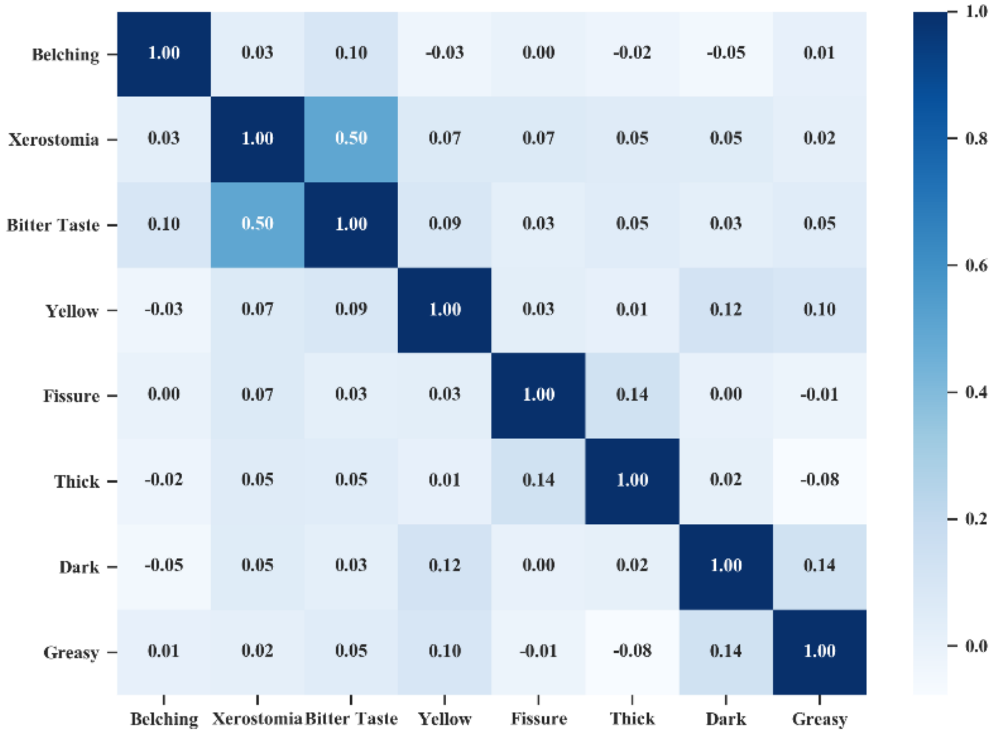


Figure S2. Correlation analysis of TDL and symptoms.

Table S3. The univariate analysis of tongue diagnostic labels in risk prediction of PLGC.

| **Characteristics** | **Pro (26) Num (Rate)** | **non-Pro (69) Num (Rate)** | **OR** | **P value** |
|---|---|---|---|---|
| Coating (Yellow) | 10(0.38) | 6(0.09) | 6.35 | 2.1e-3 |
| Greasy | 6(0.23) | 15(0.22) | 1.04 | 1.00 |
| Fissure | 5(0.19) | 8(0.12) | 1.76 | 0.56 |
| Coating (Thick) | 8(0.31) | 11(0.16) | 2.26 | 0.21 |
| Dark | 3(0.15) | 7(0.16) | 0.96 | 1.00 |

Figure S3. The operation interface of the App.

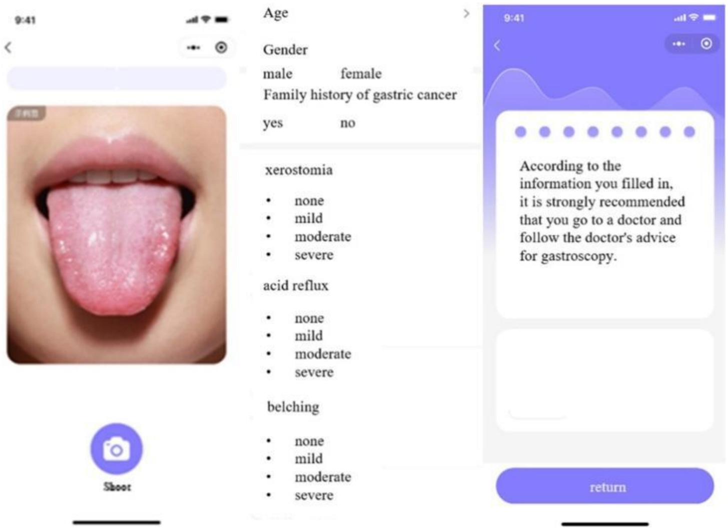

Supplement: Supplementary file 1 [file jpm-13-00271-s001.zip › jpm-2158532-supplementary.pdf]
